# Data on the pozzolanic activity in coconut shell ash (CSA) for use in sustainable construction

**DOI:** 10.1016/j.dib.2018.03.125

**Published:** 2018-03-31

**Authors:** Opeyemi Joshua, Kolapo O. Olusola, Ayobami A. Busari, Ignatius O. Omuh, Ayodeji O. Ogunde, Lekan M. Amusan, Chidiogo J. Ezenduka

**Affiliations:** a Department of Building Technology, Covenant University, Ota, Nigeria; b Department of Building, Obafemi Awolowo University, Ile-Ife, Nigeria; c Department of Civil Engineering, Covenant University, Ota, Nigeria

## Abstract

The data presented herein are results of the research summary of the investigation for pozzolanic activity in coconut shell ash (CSA) towards a sustainable construction. The data article provides information on the properties of Coconut Shell Ash that are indicative of pozzolanic activity as stated in ASTM C618-15 (2015) [1], BS EN 197-1 (2011) [2] and Joshua et al. (2018) [3]. The data are the physical property of the sand used in determining the binder strengths and the chemical and physical properties (oxide composition and Strength Activity Indices respectively) of the pulverized, calcined and sieved Coconut Shell Ash.

## Specifications Table

**Table:** 

Subject area	Engineering, Material Science, Cements and Concrete
More specific subject area	Pozzolans, Pozzolanic Cements and Blends, Concrete Strength, Cement Replacements and Sustainable Materials
Type of data	Table, graphs and figure
How data was acquired	Laboratory procedures and instrumentation to relevant standards
Data format	Raw and analyzed
Experimental factors	The agro-waste coconut shells were washed, air-dried, pulverized and calcined. The resulting ash was sieved and subjected to further laboratory tests
Experimental features	The ash was subjected to X-Ray Fluorescence (XRF) analysis. The ash also replaced cement at various percentages and the strength of the blends determined in a standard manner.
Data source location	Lagos and Ogun States, Nigeria
Data accessibility	The data are available within this article
Related research article	This is a direct submission to Data in Brief. Related research is Joshua, O., Olusola, K.O., Ogunde, A., Ede, A.N., Olofinnade, O. M., & Nduka, D. (2017). Investigating for Pozzolanic Activity in Palm Kernel Nut Waste Ash (PKNWA) with Cement towards a Sustainable Construction. International Journal of Applied Engineering Research, 12(23), 13959–13965.

## Value of the data

• This data will contribute to other research outcome that attempts to replace cement to reduce the negative impact cement pose to the environment. Cement is classed as a non-green and non-environmental friendly material that contribute to global warming and other ills associated with it [3], [4].
• This data will contribute to other materials that can be used to produce more durable concrete due to the advantage pozzolanic effect imputes to concrete durability.
• This data contributes to the list of materials that are otherwise wastes (agro-waste) that is recycled to better use in concrete production (wealth).
• This data may be helpful in the manufacturing of commercially sustainable building products.

## 1 Data

One of the indices used in measuring the level of development of a nation is the volume and level of specification of buildings and other civil structural facilities within that nation. New York and other cities in the United States, Dubai in the United Arab Emirates (thriving tourism industry) and other major cities in the world are characterized by multiple high-rise buildings that are interconnected by roads, bridges and other civil facilities. The major materials used in these buildings and facilities are Portland cement concrete and other cement based-materials. Over 90% building structures in Nigeria are made of reinforced concrete [5]. This makes concrete the world most consumed man-made materials [6]. Portland Cement (PC) is the binding material in concrete while the other materials are fillers and enhancers [7], [8]. This translates to high demand on PC while the other materials occur naturally in abundance and are used in their natural states with little or no processing.

PC is a non-environmentally friendly material due to its very high greenhouse gas (carbon dioxide, CO_2_) emission, this is a major factor in global warming with negative worldwide consequences. 5% of CO_2_ global emission occurs in concrete production with the manufacture of cement being the major contributing factor [9]. PC is an expensive material which further translates to the cost of housing. There are many successful researchers that have partially replaced cement with greener materials like pozzolans. These pozzolans occur naturally or from agricultural and industrial wastes. They are cheaper and reduce landfill contents by recycling them into concrete [3], [6]. Concrete made from cement blended with pozzolans are more durable even in harsher service environments as in phosphate and sulphate environments [10]. The data presented herein can be a tool to assess the pozzolanic activity of coconut shell as a possible partial cement replacement.

## 2 Experimental design, materials, and methods

The materials used in generating these data are the pulverized, calcined and sieved coconut shell ash (CSA), 42.5 N Dangote cement brand, drinkable borehole water and sand prepared to satisfy the standard sand specified in [11], [12]. All these materials were gotten from Ota in Ogun State and some of the coconut shell from Badagry in Lagos State Nigeria. The CSA was heated in a kiln to over 700 °C and maintained for three hours and the resulting ash was sieved through a 75 µm sieve.

The tests wherein the data was generated are the setting times on the 0% and 15% blends of CSA with cement and mortar strength performed according to [11] with cement blends of 0–25% replacement with CSA in steps of 5%. The 7-day and 28-day compressive strengths were determined to check the Strength Activity Index (SAI) and possible pozzolanic reaction between the CSA and cement. The 0% replacement, that is the 100% Portland cement, was used as a control in this experiment. Ponding method of curing was adopted with total immersion in a curing tank.

The standard sand in accordance to [11], [12] was locally prepared by washing a river-dredged sharp sand and sieved with a sieve sizes 2 mm and retained in 75 µm.

The chemical analysis of the CSA and the cement used was also determined using X-Ray *fluorescence* (*XRF*) spectrometer to evaluate for possible pozzolanic activity and classification of the CSA (see Table 1).

**Table 1:** Percentage oxide composition of the coconut shell ash (CSA) and cement.

Oxide composition	SiO_2_	Al_2_O_3_	Fe_2_O_3_	CaO	MgO	P_2_O_5_	K_2_O	SO_3_	TiO_2_	Mn_2_O_3_	SiO_2_ + Al_2_O_3_ + Fe_2_O_3_	LoI
Percentage % CSA	66.32	8.79	5.35	6.25	0.87	0.51	3.26	0.69	0.83	0.12	80.64	4.28
Percentage % Cement	15.99	4.60	2.87	58.86	2.15	0.36	0.26	0.22	0.24	0.05	–	9.63

*Where SiO_2_ is Silica oxide, Al_2_O_3_ is Aluminum oxide, Fe_2_O*_*3*_*is Iron trioxide, CaO is Calcium oxide, MgO is Magnesium oxide, P_2_O_5_ is Phosphorus oxide, K_2_O is Potassium oxide, SO_3_ is Sulphur trioxide, TiO_2_ is Titanium oxide, Mn*_*2*_*O*_*3*_*is Manganese oxide and LoI is Loss on Ignition*.

**Fig. 1:**
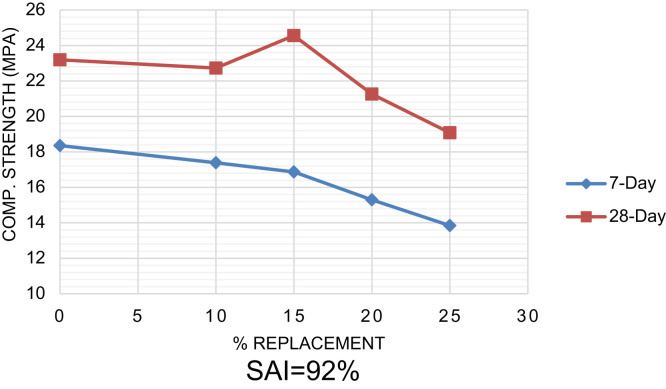
Strength development with increasing cement replacement with CSA.

The strength characteristics were determined to observe for possible pozzolanic activity and the determination of the Strength Activity Index (SAI), see Fig. 1. Setting times were carried out on the optimum replacement alone (Table 2).

**Table 2:** Setting times of the control and 10% replacement with CSA.

% of replacement with CSA	Setting time (min)	
	Initial	Final
0 (Control)	125	350
15	135	375

According to [1], [2], a pozzolan is of Class ‘*N*’ if the sum of SiO_2_, Al_2_O_3_, and Fe_2_O_3_ is greater than 70%, SO_3_ content is less than 4% and when LoI is less than 10%. The measure of pozzolanic activity is confirmed by the Strength Activity Index (SAI), which is the ratio of the 28-day strengths at 20% cement replacement with a pozzolan and the control (0% replacement). When pozzolans are blended to cements, the fresh mix is usually less workable that when cement alone is used at constant water/cement ratio [3].

If SAI is equal to or greater than 75%, pozzolanic activity may be inferred [1].
