# Supplementary material for: Data on the pozzolanic activity in coconut shell ash (CSA) for use in sustainable construction
Source: Data Brief. 2018 Mar 31;18:1142–5. doi: 10.1016/j.dib.2018.03.125 (PMC5996609; doi:10.1016/j.dib.2018.03.125)
Supplement: Supplementary file 1 — Supplementary material [file mmc1.docx]

Department of Building Technology.

Covenant University, Ota. Nigeria.

21^st^ February, 2018.

The Editor

Data in Brief.

Elsevier.

**Declaration of Conflict of Interest**

I hereby declare that there is no conflict of interest on the manuscript “*Dataset on Investigating for Pozzolanic Activity in Coconut Shell Ash (CSA) towards a Sustainable Construction*”. This manuscript is a direct submission to Data in Brief.

I wish to confirm that there are no known conflicts of interest

associated with this publication and there has been no significant financial support for this work that could have influenced its outcome.

I confirm that we have given due consideration to the protection of intellectual property associated with this work and that there are no impediments to publication, including the timing of publication, with respect to intellectual property. In so doing I confirm that we have followed the regulations of our institutions concerning intellectual property.

Thank you.


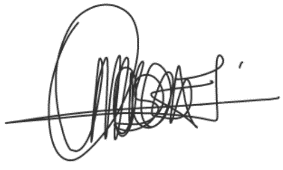


Opeyemi Joshua (Ph.D)
